# Effects of Strontium Ranelate on Spinal Interbody Fusion Surgery in an Osteoporotic Rat Model

**DOI:** 10.1371/journal.pone.0167296

**Published:** 2017-01-04

**Authors:** Tsung-Ting Tsai, Ching-Lung Tai, Natalie Yi-Ju Ho, Po-Liang Lai, Tsai-Sheng Fu, Chi-Chien Niu, Lih-Huei Chen, Wen-Jer Chen

**Affiliations:** 1 Department of Orthopaedic Surgery, Spine Section, Bone and Joint Research Center, Chang Gung Memorial Hospital and Chang Gung University College of Medicine, Taoyuan, Taiwan; 2 Graduate Institute of Medical Mechatronics, Department of Mechanical Engineering, Chang Gung University, Taoyuan, Taiwan; Universite de Liege, BELGIUM

## Abstract

Osteoporosis is a bone disease that afflicts millions of people around the world, and a variety of spinal integrity issues, such as degenerative spinal stenosis and spondylolisthesis, are frequently concomitant with osteoporosis and are sometimes treated with spinal interbody fusion surgery. Previous studies have demonstrated the efficacy of strontium ranelate (SrR) treatment of osteoporosis in improving bone strength, promoting bone remodeling, and reducing the risk of fractures, but its effects on interbody fusion surgery have not been adequately investigated. SrR-treated rats subjected to interbody fusion surgery exhibited significantly higher lumbar vertebral bone mineral density after 12 weeks of treatment than rats subjected to the same surgery but not treated with SrR. Furthermore, histological and radiographic assessments showed that a greater amount of newly formed bone tissue was present and that better fusion union occurred in the SrR-treated rats than in the untreated rats. Taken together, these results show significant differences in bone mineral density, PINP level, histological score, SrR content and mechanical testing, which demonstrate a relatively moderate effect of SrR treatment on bone strength and remodeling in the specific context of recovery after an interbody fusion surgery, and suggest the potential of SrR treatment as an effective adjunct to spinal interbody fusion surgery for human patients.

## Introduction

Osteoporosis is a metabolic disease that is characterized by the microarchitectural deterioration and loss of bone matter, eventually leading to a decrease in bone strength and an increased concomitant risk of hip, spine, and wrist fractures [1]. As it currently affects more than 200 million people and causes more than 8.9 million fractures each year worldwide, osteoporosis has become a major public health problem [2]. It is estimated that 1 out of every 3 women and 1 out of every 5 men above the age of 50 have experienced osteoporotic fractures [3]. Moreover, fractures related to osteoporosis are associated with high risks of morbidity, mortality, disability, and pain, especially for the elderly population.

Strontium ranelate (SrR), marketed as Protos by the pharmaceutical maker Servier, is an oral medication for the prevention and treatment of osteoporosis. Unlike other anti-osteoporotic drugs, SrR has a unique dual effect on both bone formation and bone resorption. It simultaneously lessens bone breakdown and stimulates bone rebuilding, preventing bone loss, improving bone strength, and reducing the risk of fractures [4].

Previous studies have proven the efficacy of SrR in reducing vertebral, nonvertebral, and hip fractures in vitro [5], in vivo [6–8], and in animal models [9–11]. However, the effects of SrR administration on interbody fusion remain unclear. The purpose of this study, then, was to determine the effects of SrR on interbody fusion in a rat tail model.

## Materials and Methods

### Animals

Twenty-four 8-week-old female Sprague-Dawley rats (weighing 200–250 g) were procured from the Laboratory Animal Center of Chang Gung Memorial Hospital and housed in environmentally controlled cages. After a 7-day quarantine period (i.e., at 9 weeks old), the rats were fed an AIN-76A diet (Research Diets Inc., New Brunswick, NJ), which is a control diet that is often used in osteoporosis-related research. AIN-76A is phytoestrogen-free and contains reduced calcium and vitamin D content, characteristics which are thought to reduce calcium absorption in animals fed with it. All the experimental protocols for this study were approved by the Institutional Animal Care and Use Committee of Chang Gung Memorial Hospital in Taoyuan, Taiwan, and were carried out in accordance with the guidelines of the National Research Council for the Care and Use of Laboratory Animals.

### Study design

When the rats were 11 weeks old, baseline measurements of bone mineral density (BMD) and bone markers were taken for each rat via, respectively, micro-CT assessment of the lumbar spine and blood sample serum analysis. After another week (i.e., at 12 weeks old), each rat underwent a bilateral ovariectomy to induce osteoporosis. Four weeks after the ovariectomy (OVX) procedure (at 16 weeks old), BMD was measured again to confirm the osteoporotic status of each rat, with confirmation being signified by a significant reduction of BMD compared to the baseline measurement; blood samples were also collected again. Then, at 17 weeks old, all the rats underwent caudal interbody fusion of the third and fourth coccygeal vertebrae of the tail. An X-ray assessment of each fusion surgery was also conducted at this point.

**Fig 1:**
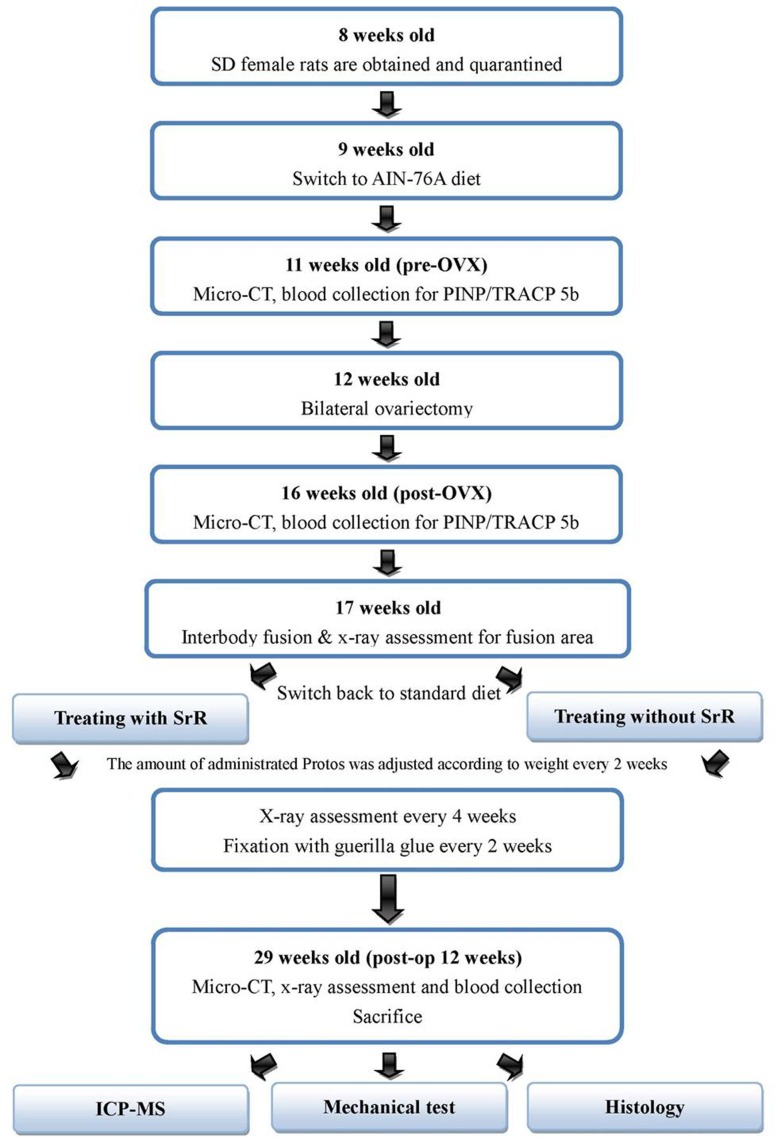
A flowchart of the study design.

Following the fusion surgery, the rats were randomly divided into two groups: a control group (n = 12) and a SrR-treated group (n = 12). At this point, because vitamin D and calcium supplements are recommended for those receiving SrR treatment, the rats in both groups were switched from the aforementioned AIN-75A diet to a standard diet (Laboratory Rodent Diet 5001; LabDiet, St. Louis, MO) containing more calcium and vitamin D content. Rats in the SrR-treated group were given 900 mg/kg/day of SrR (Protos; Servier Laboratories, Neuilly sur Seine, France) in 20 g/day of powdered standard chow, and this dose was chosen based on the study by Bruel et al. [12]. The amount of SrR administered to each rat was adjusted every two weeks according to the body weight of the rat. At 4 weeks and 8 weeks after fusion, plain radiograph assessments of the fusion status were conducted. Twelve weeks after fusion (i.e., when the rats were 29 weeks old), both plain radiograph and micro-CT assessments were conducted, and blood samples were again collected. Following the radiograph and micro-CT assessments and blood sampling, all the rats were sacrificed by pure CO_2_ exposure. After sacrifice, lumbar bone and fused tail specimens were harvested for histological and inductively coupled plasma-mass spectrometry (ICP-MS) analysis. The flowchart of the study design is presented in Fig 1.

### Surgical procedures for ovariectomy and caudal vertebral interbody fusion

All the surgical procedures were performed under inhalational anesthesia with 2% isoflurane. In addition, for each surgery, an 80-mg dose of cefazolin was injected intramuscularly and bacitracin neomycin was applied to the surgical site postoperatively to prevent infection.

For each bilateral ovariectomy, the dorso-lateral flanks of the given rat were shaved bilaterally, and the rat was placed in a prone position. A skin incision approximately 1.5 cm in length was made to expose the entrance to the peritoneal cavity, and the ovaries were pulled out by grasping the periovarian fat. The ovaries were removed. Fascia and the incision were closed with 4–0 Ethilon non-absorbable sutures.

**Fig 2:**
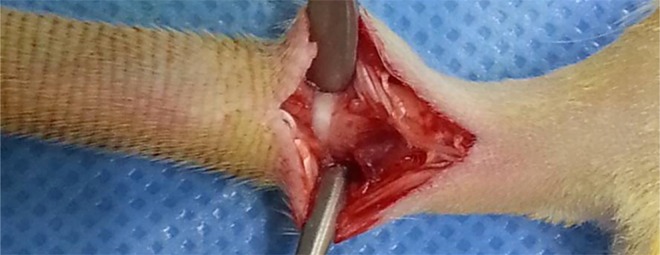
Photograph showing a rat tail following the removal of the caudal vertebral disc during the interbody fusion procedure. For each caudal interbody fusion procedure, the rat was placed in a lateral recumbent position. An incision approximately 2.5 cm in length was made in the skin of the rat’s tail, and the underlying tendons were partially removed to expose the caudal vertebrae.

For each caudal interbody fusion procedure, the rat was placed in a lateral recumbent position. An incision approximately 2.5 cm in length was made in the skin of the rat’s tail, and the underlying tendons were partially removed to expose the caudal vertebrae (Fig 2). The caudal disc between the third and fourth coccygeal vertebrae was completely removed using a rongeur. Morselized allograft from other Sprague-Dawley rats was placed in the disc space and the wound was sutured. In addition, guerilla glue, a foaming polyurethane adhesive that can function as a protective plaster cast, was applied every two weeks to the surgical site for the rats in both groups to provide support and ensure that the bones would heal in the correct position.

### Radiographic assessment

At 0, 4, 8, and 12 weeks after the fusion surgery, plain radiographs of the fused caudal vertebrae were taken and then assessed to examine the fusion status. All the radiographs were taken under the same radiographic exposure factors (penetration power: 42 kV, output current: 320 mA, distance: 120 cm and exposure time: 8 mAs). The plain radiographs were evaluated by three research fellows in a blinded fashion according to the classification system previously described in a study by Goldberg et al. [13]. Using that system, the callus maturity was classified in terms of three possible stages: stage 1, indicating nonunion; stage 2, indicating possible union; and stage 3, indicating radiographic union. The specimen was rated as non-fused if two or more evaluators graded as 1.

### Micro-CT assessment

Each rat was assessed by micro-CT 1 week before the OVX, 4 weeks after the OVX, and 12 weeks after the caudal vertebral interbody fusion surgery. After inducing general anesthesia with isoflurane and placing the given rat in a prone position, the lumbar vertebrae and fusion area of the tail were scanned using a NanoSPECT/CT scanner (BioScan Inc., Washington D.C., USA). The helical data for the lumbar region was acquired with a tube voltage of 65 kVp, an exposure time of 1000 ms, and a binning of 1:4. The helical data for the tail was acquired with a tube voltage of 45 kVP, an exposure time of 1000 ms, and a binning of 1:4. The number of projections was 240 n, with a total scan time of 8 min and 2 sec for the lumbar region and 4 min for the tail. The BMD values were measured and the images were generated using PMOD software (version 3.2; PMOD Technologies Ltd., Zurich, Switzerland).

### Serum analysis

Blood was collected from the rat tail vein 1 week before the OVX, 4 weeks after the OVX, and 12 weeks after the caudal vertebral interbody fusion surgery, and the resulting serum samples were stored at -80°C. Serum levels of procollagen type I N-terminal propeptide (PINP; a bone formation marker) and osteoclast-derived tartrate-resistant acid phosphatase form 5b (TRACP 5b; a bone resorption marker) were detected using commercially available ELISA kits from Immunodiagnostic Systems Ltd. (Fountain Hills, AZ), following the manufacturer’s protocol.

### Histological analysis

Bone and disc specimens were fixed in 10% buffered neutral formalin, decalcified in Surgipath Decalcifier II solution, embedded in paraffin, deparaffinized in xylene, and rehydrated through a series of ethanol washes. The paraffin blocks were sectioned longitudinally at a thickness of 3 μm. The sections were stained with hematoxylin and eosin and Masson’s trichrome, and then evaluated under light microscopy. The histological images were examined by three independent evaluators under the guidance of an experienced pathologist according to the grading system previously described in a study by Gordjestani et al. [14]. The fusion status of the vertebrae in each image was graded with a histological score ranging from 0 to 7, with a score of 0 indicating empty islets, a score of 1 indicating fibrosis tissue only, a score of 2 indicating more fibrosis tissue than fibrocartilage tissue, a score of 3 indicating more fibrocartilage tissue than fibrosis tissue, a score of 4 indicating fibrocartilage tissue only, a score of 5indicating more fibrocartilage tissue than bone tissue, a score of 6 indicating more bone tissue than fibrocartilage tissue, and a score of 7 indicating bone tissue only.

### Chemical analysis of calcium/strontium content

Strontium and calcium concentrations of lumbar disc, lumbar bone, and fused bone specimens were determined by ICP-MS using a SCIEX ELAN 5000 mass spectrometer (Perkin-Elmer Inc., Waltham, MA).

### Three-point bending test

As previously noted, after undergoing caudal interbody fusion, the rats were divided into two groups of 12 rats each: a control group and a SrR-treated group. Specimens from 6 rats in each of those two groups (12 rats in total) were subsequently used for the three-point bending test. For each rat, two functional units (FUs), a fusion unit and a non-fusion unit, from the rat’s tail were tested. Accordingly, a total of 24 FUs were analyzed: 12 “Non-fusion” FUs (i.e., caudal disc specimens that were not subjected to the fusion surgery) taken from both the control and the SrR-treated rats (i.e., one from each rat in both groups); 6 “Untreated fusion” FUs consisting of the 6 fused bone specimens from the control group (i.e., one from each control group rat); and 6 “Treated fusion” FUs consisting of the 6 fused bone specimens from the SrR-treated group (i.e., one from each SrR-treated group rat).

**Fig 3:**
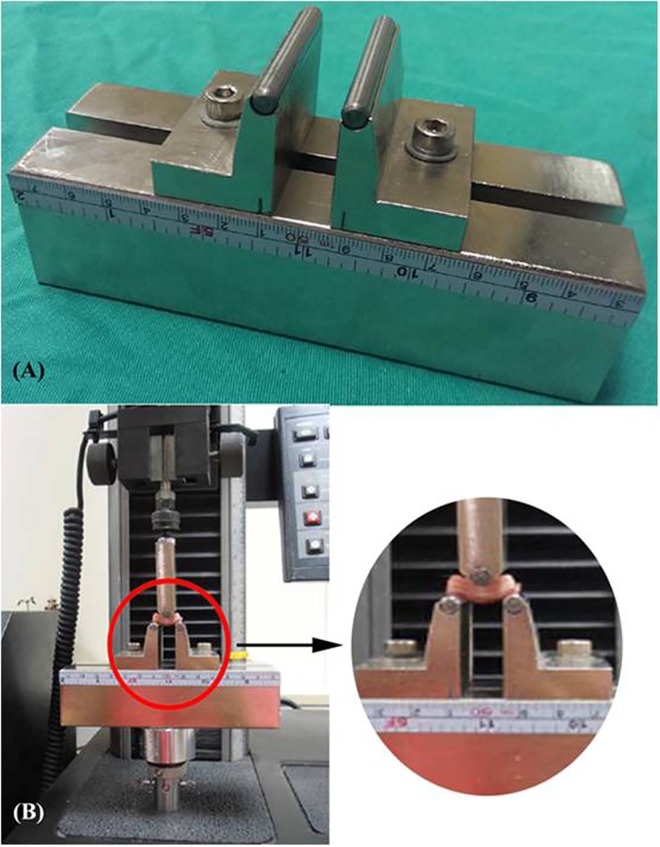
**Photographs showing (A) the steel grip used for the three-point bending test and (B) the complete experimental setup for the three-point bending test.** The FU specimen was positioned on the steel grip with a span of 18 mm, and a plunger at the mid-point of the span was clamped on the upper side of the Instron grip. An axial compressive force was applied, after positioning the construct, at a constant crosshead rate of 2 mm/min. The deflection of each FU specimen was measured to evaluate the bending stiffness for the three FU groups.

Three-point bending tests in a forward direction normal to the longitudinal axis of the rat tail were performed using an Instron testing machine (model 5544, Instron Inc., Canton, MA, USA) to compare the bending stiffness among these three FU groups. Each FU in each of the three FU groups was positioned on a steel grip with a span of 18 mm, which was itself clamped onto the lower side of the Instron frame (Fig 3A). A plunger at the mid-point of the span was clamped on the upper side of the Instron grip and connected to the load cell. An axial compressive force was applied, after positioning the construct, at a constant crosshead rate of 2 mm/min. The relationship between force and displacement (deflection) was continuously recorded in 0.05-mm increments at a sampling rate of 0.67 Hz using the Instron Merlin Software. The deflection of each FU specimen was measured to evaluate the bending stiffness for the three FU groups. The experimental set-up and testing configuration are shown in Fig 3B.

### Statistical analysis

Means and standard deviations were calculated for descriptive purposes. Multiple comparisons among the groups were performed using a one-way ANOVA test, and the comparative data between the control and SrR-treated groups were analyzed by a t-test using SigmaPlot version 10.0 (Systat Software, Inc., San Jose, CA). The results were considered significant when p-values were less than 0.05.

## Results

### Fusion results

**Fig 4:**
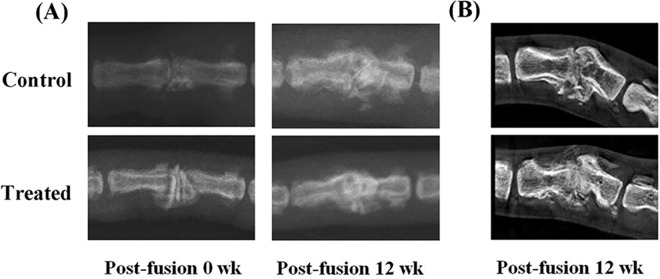
**Photographs showing (A) representative plain radiographs and (B) representative micro-CT 2D cross-section and 3D reconstruction images comparing the fusion results for the control and SrR-treated groups at 12 weeks after the interbody fusion procedure.** Better fusion union was observed in the SrR-treated group compared with the control group.

For the two control and the SrR-treated rat groups, the degree of union in the fused vertebrae was evaluated via plain radiographs and micro-CT images of the rat tails. Better fusion union was radiographically observed in the SrR-treated group compared to the control group in Fig 4. The fusion rate was 75% (9 of 12) for the control group, and 83.3% (10 of 12) for the SrR-treated group. The mean radiologic score for the SrR-treated group was 2.25±0.68, which was higher than the mean score for the control group (2.08±0.72), with no significant difference (p = 0.4028).

### Bone mineral density measurements

The osteoporotic status of the ovariectomized rats was confirmed by a significant reduction (~20%) in the bone mineral density of the fifth lumbar spine vertebrae (L5) at 4 weeks after the OVX procedure compared to before the OVX procedure. Subsequently, the mean BMD in the treated rat group was found to be higher but not statistically significant than that of the control rat group after 12 weeks of SrR treatment. Specifically, the mean BMD value of the lumbar vertebrae in the rats treated with SrR was 908.95±70.72 HU, while that of the control rats not treated with SrR was 823.63±91.75 HU. In addition, the percent changes in BMD between the different time points (that is, the percent changes from before the OVX procedure to 4 weeks subsequent to the OVX procedure, and also the percent changes from 4 weeks after the OVX to 12 weeks after the fusion surgery) for each of the two groups were also compared. From 4 weeks after the OVX to 12 weeks after the fusion surgery, the treated group showed a slight increase in BMD of 1.42±10.01%, whereas the BMD of the control group continued to decrease, falling by 11.80±7.16% over the same period. These results demonstrated that SrR treatment increased lumbar vertebral BMD after 12 weeks of treatment (Table 1).

**Table 1:** Comparison of BMD, PINP and TRACP 5b Levels for the Control Group and SrR-treated Group.

	Control (n = 12)	Treated (n = 12)	*p* value^a^
**BMD (HU)**			
Before OVX	1202.29 ± 145.00	1203.39 ± 143.43	0.989
4 wks after OVX	892.26 ± 45.24	890.43 ± 71.56	0.959
12 wks after fusion	823.63 ± 91.75	908.95 ± 70.72	0.096
**% change in BMD (%)**			
From before OVX to 4 wks after OVX	-20.36 ± 3.93	-22.49 ± 4.13	0.423
From 4 wks after OVX to 12 wks after fusion	-11.8 ± 7.16	1.42 ± 10.01	0.037*
**PINP (ng/ml)**			
Before OVX	17.38 ± 2.01	16.96 ± 2.39	0.748
4 wks after OVX	11.97 ± 1.31	12.67 ± 1.04	0.324
12 wks after fusion	8.22 ± 0.50	9.33 ± 0.80	0.041*
**% change in PINP (%)**			
4 wks after OVX	-34.10 ± 7.67	-28.03 ± 7.93	0.203
12 wks after fusion	-28.55 ± 5.76	-27.47 ± 3.97	0.713
**TRACP 5b (ng/ml)**			
Before OVX	9.04 ± 2.30	9.07 ± 2.32	0.982
4 wks after OVX	4.65 ± 0.52	4.65 ± 1.03	0.998
12 wks after fusion	2.25 ± 0.40	1.90 ± 0.53	0.186
**% change in TRACP 5b (%)**			
4 wks after OVX	-51.99 ± 7.16	-46.16 ± 5.31	0.106
12 wks after fusion	-54.63 ± 6.89	-56.88 ± 8.78	0.603

BMD, bone mineral density; OVX, ovariectomy; PINP, procollagen type I N-terminal propeptide; TRACP 5b, osteoclast-derived tartrate-resistant acid phosphatase form 5b. Values given as mean ± standard deviation.

^a^Determined by t-test comparisons of group scores.

*Significant difference between groups (p<0.05).

### Serum PINP and TRACP 5b levels

Serum values of both PINP and TRACP 5b were decreased at 4 weeks after the OVX surgery, which indicated that the OVX procedure resulted in reduced bone formation. Comparing between the control and SrR-treated groups at 12 weeks after interbody fusion, PINP level displayed a significant difference, which indicating an enhanced bone formation by strontium ranelate; however, no significant difference in TRACP 5b level was observed (Table 1).

### Histological results

**Fig 5:**
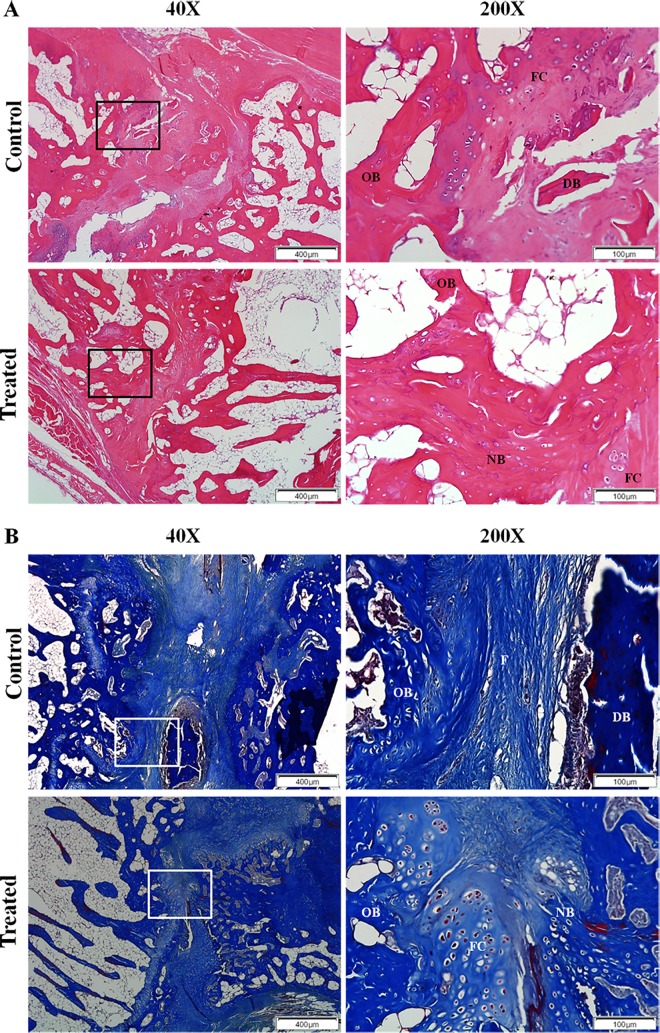
**Representative histological images of (A) H&E stain with a score of 5 for the control group and a score of 6 for the treated group, and (B) trichrome stain with a score of 4 for the control group and a score of 5 for the treated group at magnifications of 40X and 200X.** The assessment showed that, compared to the control group, a greater amount of fibrocartilage and newly formed bone tissues was present, dead bone tissue was absent, and the gap between the adjacent vertebrae also seemed to be narrower, in the SrR-treated group. OB, old bone; DB, dead bone; NB, new bone; FC, fibrocartilage; F, fibrosis.

The histological assessment showed that, compared to the control group, a greater amount of fibrocartilage and newly formed bone tissues was present, dead bone tissue was absent, and the gap between the adjacent vertebrae also seemed to be narrower, in the SrR-treated group (Fig 5). The mean histological score for the SrR-treated group (n = 6) was 5.55±0.50, which was higher than the mean score for the control group (n = 6, 4.83±0.69), with no significant difference (p = 0.0013).

### Ca/Sr concentrations

The SrR-treated rats exhibited significantly increased concentrations of strontium in both disc and bone specimens after 12 weeks of treatment (Table 2). These results confirm that SrR was indeed administered to and ingested by the treated rats. Also, there was an increase of strontium in the fused bone at the surgical site as compared to in the lumbar bones, which may suggest a beneficial effect of strontium in promoting fusion.

**Table 2:** Comparison of Calcium and Strontium Concentrations in Control Group and SrR-treated Group Disc and Bone Specimens as Determined by ICP-MS.

	Control (n = 6)	Treated (n = 6)	*p* value^a^
Calcium (ppm)			
Lumbar disc	84032.00 ± 44655.70	82179.60 ± 38341.79	0.951
Lumbar bone	124638.50 ± 15229.99	125292.00 ± 23515.19	0.967
Fused bone	137247.60 ± 17189.13	138630.00 ± 19611.89	0.914
Strontium (ppm)			
Lumbar disc	13.87 ± 7.03	916.12 ± 469.48	0.005*
Lumbar bone	67.36 ± 83.90	1740.70 ± 715.63	0.005*
Fused bone	24.65 ± 3.66	3102.56 ± 1308.75	0.001*
Ca/Sr (%)			
Lumbar disc	0.0172 ± 0.0016	1.1437 ± 0.2866	4.95E-05*
Lumbar bone	0.0518 ± 0.0631	1.4161 ± 0.5906	4.83E-03*
Fused bone	0.0179 ± 0.0011	2.2854 ± 0.9528	9.55E-04*

ICP-MS, inductively coupled plasma-mass spectrometry; Sr, strontium; Ca, calcium. Values given as mean ± standard deviation.

^a^Determined by t-test comparisons of group scores.

*Significant difference between groups (p<0.01).

### Mechanical testing results

**Fig 6:**
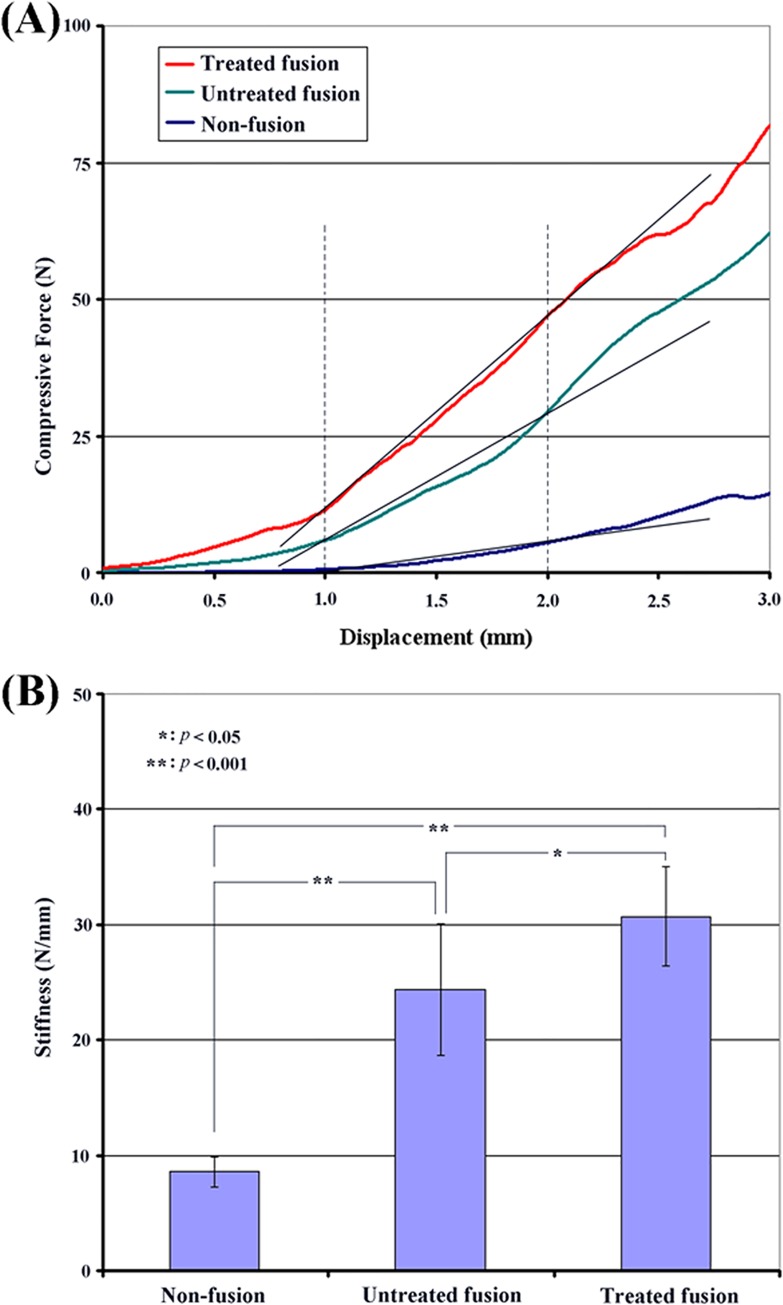
**(A) The force versus displacement curves for the three FU groups in the three-point bending test and (B) a graphical comparison of the mean bending stiffness values of the three FU groups.** Compared to the non-fusion FU group, the untreated and treated FU groups both exhibited significantly enhanced bending stiffness (p < 0.001). Furthermore, the treated FU group exhibited a statistically higher bending stiffness than the untreated FU group did (p < 0.05). These results indicate that interbody fusion surgery may improve the bending stiffness of FUs, while the bending stiffness of postoperative fusion FUs treated with SrR may be further enhanced compared to that of untreated fusion FUs.

Fig 6A shows representative force vs. displacement curves for the three functional unit (FU) groups (i.e., the “Non-fusion,” “Untreated fusion,” and “Treated fusion” groups) for the three-point bending test. For all three FU groups, a lower increasing rate of force was found at the initial phase. This might have been due to the presence of soft tissue in the FU specimens. In order to exclude the effects of such soft tissue, bending stiffness was arbitrarily defined, for each FU group, as the slope of the straight line connecting the two force values required to cause, respectively, 1.0 mm and 2.0 mm of displacement. Based on the definition, the mean bending stiffness values for the non-fusion FUs, untreated fusion FUs, and treated fusion FUs were 8.58±1.31 N/mm, 24.37±5.71 N/mm, and 31.51±4.29 N/mm, respectively (Fig 6B). Compared to the non-fusion FU group, the untreated and treated FU groups both exhibited significantly enhanced bending stiffness (*p* < 0.001). Furthermore, the treated FU group exhibited a statistically higher bending stiffness than the untreated FU group did (*p* < 0.05). Taken together, these results indicate that interbody fusion surgery may improve the bending stiffness of FUs, while the bending stiffness of postoperative fusion FUs treated with SrR may be further enhanced compared to that of untreated fusion FUs.

## Discussion

Osteoporosis already constitutes a major global health problem, and is likely to become even more prevalent and burdensome in developed countries as their populations age. Relatedly, both spondylolisthesis and degenerative spinal stenosis are being diagnosed with increasing frequency as both life expectancy and osteoporosis prevalence increase [15–16]. Spinal fusion surgery is frequently performed on osteoporotic patients with these forms of spinal instability because various studies have reported good outcomes for the procedure [17–19]. On the other hand, a failed fusion surgery can result in negative clinical outcomes [20], and in spite of continuous improvements in surgical techniques and instrumentation, fusion failure still occurs in a small but significant percentage of cases [21].

Low bone density is one potential cause of fusion failure [22], but of course, osteoporosis itself is characterized, as noted above, by the deterioration and loss of bone matter and reduced bone strength. As such, in cases requiring spinal fusion, surgeons typically utilize a variety of strategies and medications to counteract the direct effects of osteoporosis, thereby promoting positive bone remodeling and, in turn, successful fusion and better clinical outcomes.

As noted above, strontium ranelate is unique among anti-osteoporotic drugs in that it simultaneously reduces bone breakdown by curbing the activity of osteoclasts while stimulating bone rebuilding by stimulating the activity of osteoblasts. As such, the administration of SrR could potentially promote positive bone remodeling after an interbody fusion surgery. However, while a number of past studies have already demonstrated the efficacy of SrR in reducing vertebral, nonvertebral, and hip fractures in vitro [5], in vivo [6–8], and in animal models [9–11], the effects of SrR administration on interbody fusion have yet to be sufficiently investigated.

Previous publications have studied the effect of strontium ranelate on bone healing using different fracture models. Cebesoy et al. radiographically and histopathologically examined the callus maturity and bone union of closed tibial fractures in normal rats with and without strontium ranelate treatment [23], and they failed to show any effects, either beneficial or harmful, of strontium ranelate on fracture healing. However, Ozturan et al. suggested that strontium ranelate treatment achieved better fracture healing in osteoporotic rats [24]. According to radiographic assessments, biomechanical testing, and histological analysis, the treated group in their study exhibited in higher callus maturity, higher mechanical strength/fracture stiffness, and more mature woven bone after open tibial fracture than the control group.

The present study sought to quantify the effects of SrR on interbody fusion in a rat tail model. We have chosen to treat the rats with a dose of 900 mg/kg/day SrR based on previous studies that demonstrated improved bone mechanical properties in intact rats treated with this dosage [25], and the SrR concentrations in serum reported in Bruel et al. [12] are similar as the ones in postmenopausal women treated with 2 g SrR a day, a clinically relevant dosage [6]. In recent years, the caudal discs have been increasingly used for investigating the effect of drug treatment and growth factor supplementation [26–28]. Keorochana et al. [29] demonstrated a disc degeneration model at rat tail using needle puncture and claimed that the rat caudal discs are suitable for studying since it is similar as human intervertebral discs and it is simple and easily available and inexpensive. In comparison to untreated osteoporotic rats subjected to interbody fusion, SrR-treated osteoporotic rats subjected to the same fusion surgery had significantly higher lumbar vertebral BMD after 12 weeks of treatment. In addition, the treated fusion FUs of the SrR-treated rats exhibited significantly higher bending stiffness than the untreated fusion FUs of the control group rats not treated with SrR. Finally, and perhaps most critically, histological and radiographic assessments showed that a greater amount of newly formed bone tissue and better fusion union was present in the SrR-treated group compared to the control group. In short, the results of this study amply demonstrate the positive effects of SrR treatment on bone strength, remodeling, and mineral density in the specific context of recovery after an interbody fusion surgery. To our knowledge, this study is the first to address the specific question of how strontium ranelate treatment affects bone healing after spinal interbody fusion in an osteoporotic rat model.

The main limitation of this study is that there are mechanical and geometrical differences between human intervertebral discs and rat caudal discs. For example, the caudal discs of rat tails, unlike human intervertebral discs, do not experience compressive loads resulting from muscle activity during ambulation. Due to these differences between the species, the results from this animal model study are by no means fully generalizable to humans. That being said, there are, of course, various benefits to the studies involving rat models, including lower cost, the high availability of the animals for use in research, and their relatively short lifespans and rapid generational turnover. These advantages made the use of a rat model appropriate for the purposes of this study. In addition, the sample size for this study was relatively small, which may lack of sufficient statistical significance.

In conclusion, because the current study used a rat model, future studies involving osteoporotic human patients will be needed to confirm that SrR treatment can be of clinical value to patients who have undergone or are preparing to undergo a spinal fusion surgery. In addition, while the present study indicates that SrR may be more effective in promoting successful fusion union than no treatment at all, future studies should be conducted to determine whether or not SrR is more effective at promoting fusion union than alternative pharmacotherapeutic agents for osteoporosis such as bisphosphonates, statins, and various growth factors [30]. Finally, while additional research will be required to answer the above questions, the results of the present study show significant differences in bone mineral density, PINP level, histological score, SrR content and mechanical testing when comparing between the SrR-treated and control groups, and suggest the potential of strontium ranelate treatment as an effective adjunct to spinal interbody fusion surgery.

## References

[1] Osteoporosis prevention, diagnosis, and therapy. 2000. NIH consensus statement. 2000 17: 1–45. 11525451

[2] Sherman S. Preventing and treating osteoporosis: strategies at the millennium. Ann N Y Acad Sci. 2001;949: 188–197. 11795353

[3] Johnell O, Kanis JA. An estimate of the worldwide prevalence and disability associated with osteoporotic fractures. Osteoporos Int. 2006;17: 1726–1733. 10.1007/s00198-006-0172-4 16983459

[4] Marie PJ. Strontium ranelate: a dual mode of action rebalancing bone turnover in favour of bone formation. Curr Opin Rheumatol. 2006;18 Suppl 1: S11–15. 16735840 10.1097/01.bor.0000229522.89546.7b

[5] Bonnelye E, Chabadel A, Saltel F, Jurdic P. Dual effect of strontium ranelate: stimulation of osteoblast differentiation and inhibition of osteoclast formation and resorption in vitro. Bone. 2008;42: 129–138. 10.1016/j.bone.2007.08.043 17945546

[6] Meunier PJ, Roux C, Seeman E, Ortolani S, Badurski JE, Spector TD, et al The effects of strontium ranelate on the risk of vertebral fracture in women with postmenopausal osteoporosis. N Engl J Med. 2004;350: 459–468. 10.1056/NEJMoa022436 14749454

[7] Reginster JY, Felsenberg D, Boonen S, Diez-Perez A, Rizzoli R, Brandi ML, et al Effects of long-term strontium ranelate treatment on the risk of nonvertebral and vertebral fractures in postmenopausal osteoporosis: Results of a five-year, randomized, placebo-controlled trial. Arthritis Rheum. 2008;58: 1687–1695. 10.1002/art.23461 18512789

[8] Seeman E, Devogelaer JP, Lorenc R, Spector T, Brixen K, Balogh A, et al Strontium ranelate reduces the risk of vertebral fractures in patients with osteopenia. J Bone Mineral Res. 2008;23: 33–38. 10.1359/jbmr.071105 17997711

[9] Bain SD, Jerome C, Shen V, Dupin-Roger I, Ammann P. Strontium ranelate improves bone strength in ovariectomized rat by positively influencing bone resistance determinants. Osteoporo Int. 2009;20: 1417–1428. 10.1007/s00198-008-0815-8 19096745

[10] Farlay D, Boivin G, Panczer G, Lalande A, Meunier PJ. Long-term strontium ranelate administration in monkeys preserves characteristi cs of bone mineral crystals and degree of mineralization of bone. J Bone Miner Res. 2006;20: 1569–1578. 10.1359/JBMR.050405 16059629

[11] Marie PJ, Hott M, Modrowski D, De Pollak C, Guillemain J, Deloffre P, et al An uncoupling agent containing strontium prevents bone loss by depressing bone resorption and maintaining bone formation in estrogen-deficient rats. J Bone Miner Res. 1993;8: 607–615. 10.1002/jbmr.5650080512 8511988

[12] Bruel A, Olsen J, Birkedal H, Risager M, Andreassen TT, Raffalt AC. Strontium is incorporated into the fracture callus but does not influence the mechanical strenght of healing rat fractures. Calcif Tissue Int. 2011;88: 142–152. 10.1007/s00223-010-9439-z 21153023

[13] Goldberg VM, Powell A, Shaffer JW, Zika J, Bos GD, Heiple KG. Bone grafting: role of histocompatibility in transplantation. J Orthop Res. 1985;3: 389–404. 10.1002/jor.1100030401 3906062

[14] Gordjestani M, Dermaut L, De Ridder L, De Waele P, De Leersnijder W, Bosman F. Digital measurements: a different approach to evaluate bone formation. A technical report. Cells Tissues Organs. 2006;184: 148–153. 10.1159/000099621 17409740

[15] Szpalski M, Gunzburg R. Lumbar spinal stenosis in the elderly: an overview. Eur Spine J. 2003;12: S170–175. 10.1007/s00586-003-0612-1 13680315 PMC3591819

[16] Yone K, Sakou T, Kawauchi Y, Yamaguchi M, Yanase M. Indication of fusion for lumbar spinal stenosis in elderly patients and its significance. Spine. 1996;21: 242–248. 8720411 10.1097/00007632-199601150-00016

[17] Cavagna R, Tournier C, Aunoble S, Bouler JM, Antonietti P, Ronai M, et al Lumbar decompression and fusion in elderly osteoporotic patients: a prospective study using less rigid titanium rod fixation. J Spinal Disord Tech. 2008;21: 86–91. 10.1097/BSD.0b013e3180590c23 18391710

[18] Glassman SD, Polly DW, Bono CM, Burkus K, Dimar JR. Outcome of lumbar arthrodesis in patients sixty-five years of age or older. J Bone Joint Surg Am. 2009;91: 783–790. 10.2106/JBJS.H.00288 19339561

[19] Sienkiewicz PJ, Flatley TJ. Postoperative spondylolisthesis. Clin Orthop Relat Res. 1997;(221): 172–180. 3608297

[20] Okuda S, Miyauchi A, Oda T, Haku T, Yamamoto T, Iwasaki M. Surgical complications of posterior lumbar interbody fusion with total facetectomy in 251 patients. J Neurosurg Spine. 2006;4: 304–309. 10.3171/spi.2006.4.4.304 16619677

[21] Bridwell KH, Sedgewick TA, O'Brien MF, Lenke LG, Baldus C. The role of fusion and instrumentation in the treatment of degenerative spondylolisthesis with spinal stenosis. J Spinal Disord. 1993;6: 461–472. 8130395 10.1097/00002517-199306060-00001

[22] Coe JD, Warden KE, Herzig MA, McAfee PC. Influence of bone mineral density on the fixation of thoracolumbar implants. A comparative study of transpedicular screws, laminar hooks, and spinous process wires. Spine. 1990;15: 902–907. 2259978 10.1097/00007632-199009000-00012

[23] Cebesoy O, Tutar E, Kose KC, Baltaci Y, Bagci C. Effect of strontium ranelate on fracture healing in rat tibia. Joint Bone Spine. 2007;74: 590–593. 10.1016/j.jbspin.2007.01.034 17967557

[24] Ozturan KE, Demir B, Yucel I, Cakıcı H, Yilmaz F, Haberal A. Effect of strontium ranelate on fracture healing in the osteoporotic rats. J Orthop Res. 2011;29: 138–142. 10.1002/jor.21204 20726035

[25] Ammann P, Shen V, Robin B, Mauras Y, Bonjour JP, Rizzoli R. Strontium ranelate improves bone resistance by increasing bone mass and improving architecture in intract female rats. J Bone Miner Res. 2004;19: 2012–2020. 10.1359/JBMR.040906 15537445

[26] Zhang H, La Marca F, Hollister SJ, Goldstein SA, Lin CY. Developing consistently reproducible intervertebral disc degeneration at rat caudal spine by using needle puncture. J Neurosurg Spine. 2009:10: 522–30. 10.3171/2009.2.SPINE08925 19558284

[27] Sato J, Sakuma Y, Yamauchi K, Orita S, Kubota G. Oikawa Y, et al Elevated VEGF in degerative intervertebral discs in rats with injured intervertevral discs of the caudal vertebrae. Global Spine J. 2014;04: 165.

[28] Walsh AJ, Bradford DS, Lotz JC. In vivo growth factor treatment of degenerated intervertebral discs. Spine. 2004;29: 156–163. 10.1097/01.BRS.0000107231.67854.9F 14722406

[29] Keorochana G, Johnson JS, Taghavi CE, Liao JC, Lee KB, Yoo JH, et al The effect of needle size inducing degeneration in the rat caudal disc: Evaluation using radiograph, magnetic resonance imaging, histology, and immunohistochemistry. Spine J. 2010;10: 1014–1023. 10.1016/j.spinee.2010.08.013 20970740

[30] Lane NE, Kelman A. A review of anabolic therapies for osteoporosis. Arthritis Res Ther. 2003;5: 214–222. 10.1186/ar797 12932280 PMC193734

